# Measurement of bilirubin in cerebrospinal fluid using the oxidase method on automated chemistry system advia XPT

**DOI:** 10.1016/j.plabm.2025.e00473

**Published:** 2025-04-25

**Authors:** Ida Branzell, Gabriella Lillsunde Larsson, Dieter Samyn, Paul Pettersson-Pablo

**Affiliations:** a Department of Laboratory Medicine, Clinical Chemistry, Örebro University Hospital, Örebro, Sweden; b School of Health Sciences, Örebro University, SE-701 82 Örebro, Sweden; c Department of Laboratory Medicine, Faculty of Medicine and Health, Örebro University Hospital, Örebro, Sweden

**Keywords:** Bilirubin, Cerebrospinal fluid, Spectrophotometry, Siemens chemistry Advia XPT, Subarachnoid haemorrhage, Clinical laboratory

## Abstract

**Background and aim:**

Evaluate the diagnostic performance of automated, quantitative bilirubin measurement, modified to extend its lower measurement ranges, in cerebrospinal fluid (CSF) using the Siemens analyzer Advia XPT. Results were compared with the gold standard spectrophotometry for diagnosis of subarachnoid haemorrhage (SAH).

**Method:**

Eighty clinical samples were analyzed on an Advia XPT, and results were compared to spectrophotometric results using the Agilent Cary 100 bio system. Method performance at low concentrations were evaluated using diluted control material and patient plasma and CSF samples. ROC curve analysis determined a suitable cutoff.

**Result:**

Evaluation of low-concentration performance, below 2 μmol/L on Advia XPT, showed a measurement bias of -1.0 %, and a linear regression equation of y = 0.843x + 0.0351 (R^2^ of 0.975), describing the relationship between measured and expected concentrations of diluted samples. The coefficient of variation, (CV), was 2.92 % at 0.598 μmol/L and 26.6 % at 0.161 μmol/L. Using the outcome of the analysis on Agilent Cary 100 as reference, sensitivity was 100 % and specificity 96 %, employing a cutoff of 0.41 μmol/L.

**Conclusion:**

Quantitative measurement of bilirubin in CSF using the bilirubin oxidase method on the automated Advia XPT platform perform well, with the analysis of low concentrations of bilirubin displaying a high precision and a high concordance with the results of spectrophotometry. These preliminary findings are indicative of the merits of quantitative measurement, that warrants further study of its diagnostic potential as an alternative to the more cumbersome spectrophotometry for diagnosing SAH.

## 1 Background

Normally, bilirubin is present only at low or undetectable concentrations in cerebrospinal fluid (CSF). However, elevations occur in patients with a subarachnoid haemorrhage (SAH). During a haemorrhage the red blood cell count in CSF increases and the cells begin to lyse, which in turn generates free hemoglobin. The free hemoglobin is metabolized to bilirubin, that is detectable as an elevated bilirubin and guidelines recommend it is measured along with oxyhaemoglobin, for the diagnosis of SAH [[1], [2], [3]].

Computed tomography (CT) is the first line diagnostic modality used in case of a suspected SAH. However, its sensitivity for detecting haemorrhage drops to 50 % one week after the onset of symptoms and approaches zero percent after three weeks [4]. In patients where the CT is negative, but a diagnosis of SAH is still considered likely, a biochemical analysis of CSF is warranted. However, no international consensus is developed for SAH diagnosis, and different countries, regions and hospitals follow separate protocols for handling patients with suspected SAH. The choice of what analyses to perform on the CSF also differs between hospitals [5]. Currently, spectrophotometric measurement of bilirubin is regarded as the gold standard biochemical analysis of CSF for diagnosing SAH [[6], [7], [8]].

The employment of a highly automated chemistry instrument, typically available at most clinical laboratories, has been put forth as an alternative to spectrophotometry. Since CSF normally contains a much lower concentration of bilirubin compared to serum, the serum method usually needs modification to expand the linearity downwards to permit an analysis of lower concentrations [9,12]. When analyzing the bilirubin concentration in serum, two different methods are typically used, the diazo method and the oxidase method [10,11]. Briefly, the diazo method involves the reaction of bilirubin with a diazonium salt at acidic pH. While cost-effective and easily automated, it can be prone to interference from hemolysis [10]. The enzymatic oxidase methods, such as vanadate oxidase, oxidize bilirubin to biliverdin at acidic pH, are less sensitive to hemolytic interference [11]. The diazo method has previously been studied within the context of CSF bilirubin [9,12], but to our knowledge, there are currently no publications on using the oxidase method.

In this study, we evaluated the performance of quantitative measurement of total bilirubin in cerebrospinal fluid using the oxidase method on samples from patients with a suspected subarachnoid haemorrhage and compared the results to those obtained using spectrophotometry.

## 2 Method and Materials

Ethical approval was obtained from Swedish Ethical Review Authority, ref no 2020-06574.

### 2.1 Sample collection and storage

All CSF samples included in this study were collected in conical sample tubes made of polypropylene (Sarstedt AB, Nümbrecht, Germany) and protected from light using aluminum foil. Upon arrival at the laboratory, the samples were centrifuged (Rotana 460 R, Hettich GmbH, Tuttlingen, Germany) for 7 min at 2400 g and the supernatant was transferred to a 5 ml tube (Sarstedt AB, Nümbrecht, Germany) and thereafter transferred to a cryo tube (VWR International, Spånga, Sweden) and stored at -80 °C. Only samples referred to the laboratory for spectrophotometric analysis for suspected SAH were included in the study. 80 CSF samples were included in total. The first sample was collected in 2012 and the last sample was collected in 2019. Consequently, the age of the samples analyzed varied between only a few hours up to 8 years.

50 out of the 80 CSF samples had an associated serum sample arriving at the same time to the laboratory along with the CSF. These samples were centrifuged (Rotana 460 R, Hettich GmbH, Tuttlingen, Germany) for 7 min at 2400 g before undergoing analysis of serum bilirubin concentration.

### 2.2 Agilent Cary 100

Analysis on Agilent Cary 100 bio (Agilent, Santa Clara, USA) (Cary), generates an absorbance spectrum between the wavelengths of 350 to 650 nm. The software for the analysis is provided by Agilent. The operator manually draws a baseline, roughly between 360 and 530 nm, depending on the characteristics of the spectrum. The program automatically calculates the Net Oxyhaemoglobin Absorbance (NOA) and Net bilirubin absorbance (NBA). Absorbance spectras were considered negative, according to UK NEQAS guidelines, when NBA was ≤0.007 AU and NOA ≤0.02 AU. Results were obtained from the Cary, based on the original scans upon arrival at the laboratory (at the time of patient presentation at the emergency clinic). The samples were subsequently frozen and analyzed on Siemens Advia XPT.

### 2.3 Siemens Advia XPT

During the study, the software version used with Advia XPT was V.1.1. A modification to the original method was made, with the purpose of obtaining a measurement range that extends into lower concentrations, from the default of 2 - 600 μmol/L to 0 - 600 μmol/L. The method was calibrated using a single point calibrator with distilled water used as a blank.

The method for measuring total bilirubin in serum on Siemens Chemistry Advia XPT is an oxidase-method utilizing vanadate (VO^3−^) to catalyze the oxidation process. According to the manufacturer, the analysis is linear between 2 and 599 μmol/L [13]. It is traceable to the American association for clinical chemistries (AACC) reference method, which uses material from National institute of standards, and technology (NIST) [14].

The measured AU at 476 nm on Cary was converted to a bilirubin concentration in μmol/L, based on a described comparative molar absorptivity of 0,000042 [23] to enable a quantitative comparison between spectrophotometry and biochemical measurement for calculating the bias, to enable the calculation of bias and correlation between bilirubin concentration on Advia XPT to measured AU on Cary.

### 2.4 Serial dilutions evaluation

Three plasma samples, with original concentrations 5.8, 12 and 26 μmol/L, respectively, were each separately serially diluted with sodium chloride (NaCl) 0.9 %, with the purpose of generating a series of low concentration datapoints at a range that could be expected to encompass concentrations that are typically observed in cerebrospinal fluid, starting from 2 μmol/l (the manufacturer’s lower limit of linearity) extending into lower concentrations. This generated a total of 22 points, ranging from expected values of 0.075 to 2.6 μmol/L. All samples were measured in duplicate, using the mean of the two results to evaluate the method performance at low concentrations. The results were evaluated using linear regression. A trend line was fitted to examine the relationship and possible measurement bias between measured and calculated concentrations.

### 2.5 Repeatability

To evaluate method repeatability at low concentrations, commercial controls and patient CSF samples were analyzed. The analysis was performed in a single run. Control samples (Bio-Rad Liquid Unassayed Multiqual 1, Hercules, USA) were diluted to five different levels with NaCl (0.9 %), and two of the CSF samples were analyzed ten times each on ADVIA XPT. Three CSF pools of patient samples were created. Two of the pooled patient samples were analyzed 20 times, the third analyzed only 19 times, due to a shortage of sample. Repeatability on Cary was examined by analyzing the same sample 10 times and reported as CV%.

### 2.6 Accuracy

The assessment of method accuracy, comparing the new method on Advia XPT to the absorption spectrum on Cary, was performed in accordance with UK Guidelines [8]. A total of 80 patient samples were analyzed on both instruments. All samples were analyzed once on Cary and analyzed twice on Advia XPT to enhance the reliability of the data obtained from this platform and minimize the impact and risk of random measurement error.

### 2.7 Statistics

The following statistics softwares were used in this study: the plug-in program Analyze-it (Analyze-it Software, Ltd., Leeds, United Kingdom) was used with Microsoft Excel (Microsoft Corporation, Redmond, Washington, USA) and SPSS v.25 (IBM Corp., Armonk, N.Y., USA). Descriptive statistics were presented as the coefficient of variation (CV) and the standard deviation (SD) were used to assess method repeatability. Sensitivity and specificity was calculated using the results of spectrophotometric measurement (Cary) as a reference and analyzed using a receiver operator curve (ROC-curve) to assess diagnostic performance and determine the optimal cut-off value based on our results.

## 3 Results

### 3.1 Serial dilutions evaluation

The serial dilutions in the low range were plotted in a linear regression model, to which a trend line was fitted (correlation coefficient 0.988) that corresponded to y = 0.843x + 0.0351, with an R^2^ of 0.975. The mean bias between expected and measured values across this lower range was -1.0 %.

### 3.2 Repeatability

Repeatability at low concentrations of bilirubin is shown in Table 1. The CV% of the CSF patient samples were 2.92 % (at a mean concentration 0.598 μmol/L) and 13.6 % (at a mean concentration 0.225 μmol/L). The three pools of patient samples were analyzed 19-20 times resulting in CV’s ranging from 10.8 to 12.3 %, at a mean concentration of 0.379 μmol/L, 0,395 μmol/L and 0.258 μmol/L, respectively. As seen in Table 1, the calculated CV decreased with higher concentrations of bilirubin.

**Table 1:** A summary of calculated mean concentration of bilirubin, standard deviation and coefficient of variation for the repeatability measurements performed on Siemens Advia XPT.

	Diluted Control material					Patient Sample		Pooled Patient Samples		
n	10	10	10	10	10	10	10	20	20	19
Mean (μmol/L)	0.161	0.208	0.294	0.444	0.549	0.225	0.598	0.258	0.379	0.395
SD (μmol/L)	0.043	0.036	0.049	0.041	0.030	0.031	0.017	0.032	0.041	0.048
CV (%)	26.6	17.4	16.8	9.10	5.40	13,6	2.92	12.3	10.8	12.1

### 3.3 Accuracy

**Fig. 1:**
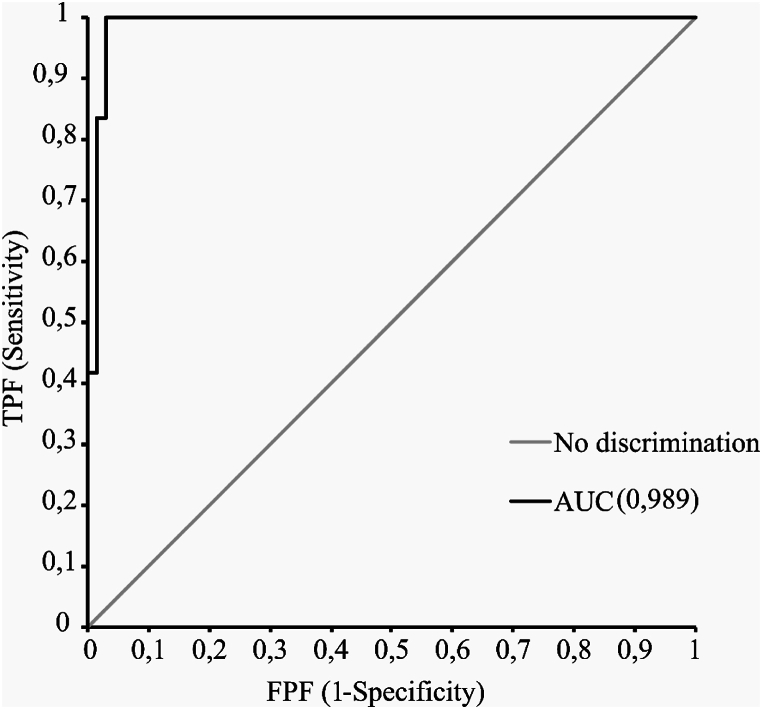
Receiver operating characteristic curve (ROC) with diagnosis based on spectrophotometric measurement (Cary) as a reference.

The ROC curve analysis (Fig. 1) gave rise to an area under the curve (AUC) of 0.989 (95 % CI 0,970 – 1.01).

**Fig. 2:**
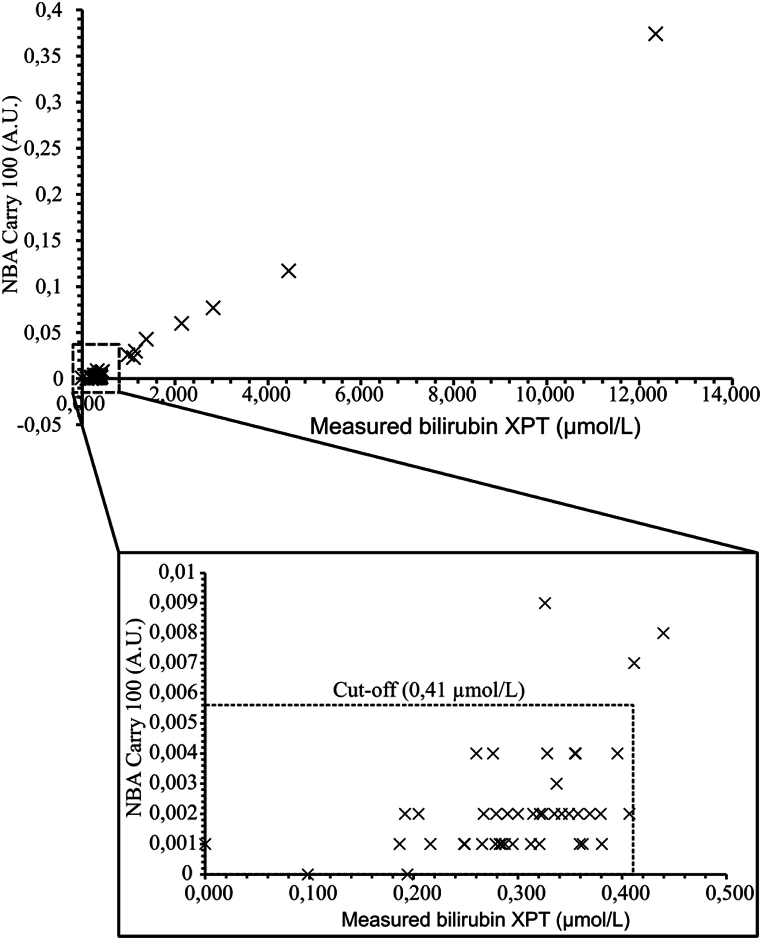
Scatterplot comparison between NBA values (in A.U.) on Agilent Cary 100™ and measured bilirubin concentrations in CSF on Siemens Advia XPT™ (in μmol/L). The scatterplot contains all obtained data points; the zoomed-in box contains data points that show a narrower range around the cut-off value (0,4 μmol/L). The cut-off value is indicated by dotted line.

Plotting the sensitivities and specificities at different cut-off values, the optimal cut-off value was determined to be 0.41 μmol/L. This concentration corresponded to 0.0055 AU on Cary (Fig. 2). At this cut-off, the method had a sensitivity of 100 % and a specificity 96 %. Three of the samples showed a discrepancy between Advia XPT and spectrophotometry results, being positives on Advia XPT and negative on Cary (Table 2). Two of the false positive samples had a concentration of 0,41 μmol/L, i.e. right on our proposed cut-off, and the third had a high bilirubin content of 2,59 μmol/L.

**Table 2:** The number of positive respectively negative results measured on Agilent Cary and Siemens Advia XPT with the cut-off 0.41 μmol.

		Cary	
		Positive	Negative
**Advia XPT**	**Positive**	12	3
	**Negative**	0	65

**Fig. 3:**
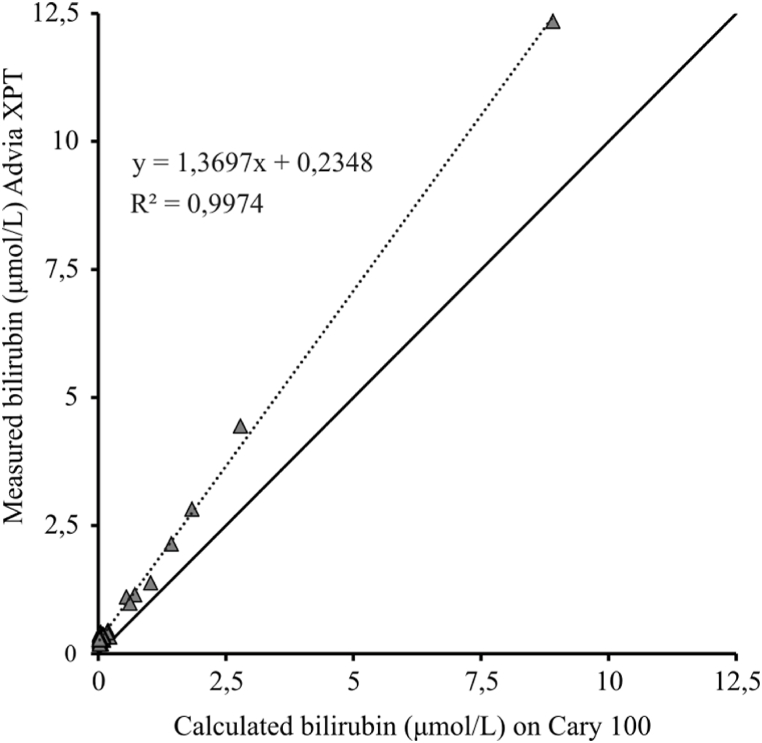
Scatterplot comparison between measured bilirubin concentrations in CSF on Siemens Advia XPT™ and the calculated concentrations on Agilent Cary 100™.

When calculating the absorbance measured on Cary from AU to μmol/L [45] and comparing the calculated concentrations of bilirubin to the measured concentrations on Advia XPT, a positive bias was observed, see Fig. 3. The correlation between the two methods is 0.999 (95 % CI 0,242 – 0,532). Thirty-two samples did not display any measurable NBA on Cary and were therefore excluded from the calculation, which left 48 samples analyzed, ranging from 0 to 41.05 μmol/L.

### 3.4 Impact of serum bilirubin

50 out of the 80 CSF samples measured in this study had an associated serum sample arriving at the same time to the laboratory along with the CSF. Out of these, no serum sample contained elevated bilirubin concentrations (mean: 11,7 μmol/L, SD: 5,0 μmol/L). The laboratory reference interval for bilirubin is <25 μmol/L [5]. The calculated correlation between measured bilirubin in serum and bilirubin in CSF was 0,027.

## 4 Discussion

In this study we evaluated the performance of the measurement of bilirubin in CSF using the oxidase method on automated chemistry analyzer Advia XPT. We consider the high concordance with the spectrophotometry results, with only three discrepant results out of 80, to show promise as an alternative to the diagnosis of SAH by spectrophotometry. Finding the optimal laboratory biomarker for diagnosing SAH has been an ongoing endeavor for over 20 years, where different analytes, with examinations of alternate biomarkers such as D-dimer and ferritin, having been investigated for their diagnostic potential as an alternative to spectrophotometry [[15], [16], [17], [18]]. Current consensus stipulates that a spectrophotometric method for analysis of bilirubin and oxyhaemoglobin, when diagnosing SAH, is recommended, based on it being well-established thanks to its high sensitivity [5]. New methods and instruments with higher sensitivity and specificity, with the capacity of quantitative measurement of the proposed biomarkers at low concentrations, warrant the exploration of alternatives to spectrophotometric analysis. Various approaches have been suggested, e.g. Beetham et al. proposed the employment of a combination of the two methods where all samples are analyzed for bilirubin concentration and only samples above the cut-off are selected for confirmative analysis using a spectrophotometry. This approach limits the frequency with which a laboratory has to perform spectrophotometric analysis, but it could also be argued that such an approach risks complicating diagnostic procedure and prolong the response time [19].

An automated analysis of bilirubin in CSF with a set cut-off value has several advantages benefitting the clinical laboratory. It is potentially more cost-effective, provides faster results that are easier to interpret, that are less operator dependent. On the other hand, quantitative concentration measurement is not as well established compared to the spectrophotometric method and analytic performance and cut-offs need to be carefully investigated and calculated for each instrument and method specifically, ideally in relation to clinical outcome data.

The limitation and possible drawback of SAH diagnosis based on quantitative measurement, and a likely explanation to its limited use, is that the accompanying measurement of oxyhaemoglobin would not be performed. This is a disadvantage with respect to current guidelines, such as the well-established UK-NEQAS guidelines, in which oxyhaemoglobin concentration measurement is part of the algorithm. An increase in CSF can be detected before the formation of bilirubin, and may thus function as an earlier biomarker of SAH; i.e. after CT begins to lose its sensitivity (but prior to the formation of bilirubin). Bilirubin based diagnostics of SAH typically requires waiting for at least 12-24 h after onset of symptoms before performing the analysis. Nonetheless, in a recent paper evaluating the performance of two different SAH diagnostic algorithms, it was noted that oxyhaemoglobin can interfere with results and give rise to false positives [20]. The presence of oxyhaemoglobin in the sample only indicates that blood has been shed into the CSF at some point and may have various causes making it less specific for SAH. Altogether, it can be argued that oxyhaemoglobin may sometimes be superfluous in the diagnosis of SAH, as long as CT is performed and the directive that lumbar puncture be performed no earlier than 12 h after symptom onset is adhered to Refs. [5,8].

### 4.1 Method performance

Advia XPT performed well when examined for its performance in measuring diluted plasma samples in the low range. Repeatability showed low CVs at low ranges of commercial controls and CSF samples. A correlation of 0.988 and R^2^ of 0.975 for measured bilirubin compared to the calculated concentration, suggests a satisfactory accuracy well below 2.0 μmol/L, to a concentration that is even lower than what is typically observed in CSF samples. One disadvantage of this study is that the serial dilution tests performed used only one specific reagent and calibrator batch. As is commonly experienced when trying out different batches, there is no guarantee that the result would be as promising using other combinations of batches. Such differences between batches must be monitored to ensure that new batches provide similar results. These results were based on the analysis of diluted plasma samples, and it would have been ideal to use clinical CSF samples for evaluating linearity, but CSF samples containing high enough concentrations of bilirubin, in combination with a large enough sample volume to allow executing a dilution series, were somewhat rare. An accurate examination of the LoQ would have been warranted but was not examined in our paper. Based on the imprecision data (Linear regression, CV% as a function of bilirubin concentration), LoQ was 0,228 μmol/L, which is where we calculated a CV% to correspond to 20 % [23]. Concentrations within this range were easily distinguishable from the blank, and still provided a somewhat comfortable margin towards our proposed diagnostic cut-off of 0.41 μmol/L (0.161 μmol/L – 26.6 % compared to 0.444 μmol/L – 9.1 %), Table 1. Ungerer et al. observed, when examining the repeatability, a CV of 7 % for a control sample at 0.400 μmol/L (n=20) [9]. The repeatability of our method had a CV of 9 % at 0.444 μmol/L on our diluted control material (Table 1). The repeatability stated here were all drawn from a single analytical run. Further investigation of the repeatability of the method, using different lots, is warranted to also evaluate the precision of the method over several days, with different calibrator batches.

### 4.2 Accuracy

The calculated cut-off of 0.41 μmol/L generated a high specificity while maintaining a sensitivity of 100 % in comparison with Cary. This specificity of 96 % is slightly higher than what Ungerer et al. reported (92 %) using an Aeroset analyzer (Abbott laboratories) [9]. In 2007, Che-Yung Chao et al. attempted to replicate Ungerer et al.’s study obtaining similar results. They employed a slightly higher cut-off (0.38 μmol/L) and obtained a sensitivity of 100 % and a specificity of 94.4 % [21]. With respect to the three falsely positive samples we observed on Advia XPT, we found that two of them straddled the cut-off, while the third contained a strongly elevated bilirubin concentration. The reason for this is unfortunately unknown, and we had no means to examine patient data further or an access to a serum sample to investigate potential causes such as contamination or storage artefacts. Of course, receiving CSF samples for SAH, that lack an accompanying serum sample, is a source of error that equally affects the interpretation of spectrophotometry measurement.

Calculating measured NBA in AU, then converting to μmol/L, to be able to compare the absorbance peak on Cary for bilirubin to the measured concentration on Advia XPT might not be a valid comparison given their differing methodology. The linear correlation between the two methods is reassuring but does not lend irrefutable support to the Advia XPT as a fully equal substitute to spectrophotometry. The interpretation of spectrophotometry yields binary results, that are either consistent or not with the diagnosis of SAH. I.e. the results are either positive or negative, not reported as a specific concentration of bilirubin. This is why we believe that calculating the sensitivity and specificity of Advia XPT is a better tool for evaluating the method’s accuracy compared to Cary.

There are important limitations to our study, in particular our inability to give a detailed assessment the impact of Serum Bilirubin. No correlation was seen between serum bilirubin and bilirubin in CSF. Unfortunately, no serum samples containing elevated levels of bilirubin were included in the study, which makes the assumption that no correlation exists based on this result precipitous. The long duration of the sample collection stage, spanning up to eight years, resulted in a great variation in storage time of the samples before analysis on Advia XPT. Samples were analyzed immediately after collection on Cary, then stored (up to eight years) before analysis was performed on Advia XPT. As the exact point in time of collection for many of the samples was not registered, we were unable to take storage time into account. Furthermore, in this pilot study we chose to evaluate quantitative measurement performance in comparison with gold standard spectrophotometry measurement, but it is possible that a proportion of the results were false positives or false negatives using spectrophotometry with the respect to the final clinical diagnosis. Further studies evaluating the performance of quantitative measurement with respect to clinical outcome are warranted.

To our knowledge, this is the first study to evaluate an oxidase method for quantitative measurement of bilirubin for SAH diagnosis in comparison with spectrophotometry. Previously performed and published studies [9,12,21] are all modifications to the diazo method while we used an oxidase method. The suggested increase to the cut-off might, in part, be explained by the difference in methodology and instrumentation. Florkowski et al. put forth a need for more studies to validate the cut-off value before committing entirely to changing the methodology [22]. The fact that we replicated the results of Ungerer et al. and Ahmed et al. further underlines the validity of quantitative measurement as an alternative to spectrophotometry. The high sensitivity (100 % compared to spectrophotometry) even at a higher cut-off is reassuring.

## 5 Conclusion

Quantitative measurement of bilirubin in CSF on Advia XPT correlates well with spectrophotometry for diagnosing SAH. A cut-off of 0.41 μmol/L corresponds to a sensitivity of 100 % and a specificity of 96 % in comparison with the results obtained using the Cary. While further inquiry into the stability, sources of error and interferences of the method are warranted, the results of this study suggest that quantitative measurement could come to be a feasible alternative to spectrophotometric diagnosis of SAH.

## CRediT authorship contribution statement

**Ida Branzell:** Writing – review & editing, Writing – original draft, Methodology, Investigation, Formal analysis, Data curation, Conceptualization. **Gabriella Lillsunde Larsson:** Writing – review & editing, Writing – original draft, Supervision, Conceptualization. **Dieter Samyn:** Writing – review & editing, Visualization. **Paul Pettersson-Pablo:** Writing – review & editing, Writing – original draft, Supervision, Conceptualization.

## Declaration of competing interest

The authors declare that they have no known competing financial interests or personal relationships that could have appeared to influence the work reported in this paper.

## Data Availability

Data will be made available on request.
